# Bragg Curve Detection of Low-Energy Protons by Radiophotoluminescence Imaging in Lithium Fluoride Thin Films

**DOI:** 10.3390/s23104779

**Published:** 2023-05-16

**Authors:** Rosa Maria Montereali, Valentina Nigro, Massimo Piccinini, Maria Aurora Vincenti, Alessandro Ampollini, Paolo Nenzi, Concetta Ronsivalle, Enrico Nichelatti

**Affiliations:** 1 Fusion and Technologies for Safety and Security Department, ENEA C.R. Frascati, Via E. Fermi 45, 00044 Frascati, Italy; rosa.montereali@enea.it (R.M.M.); valentina.nigro@enea.it (V.N.); massimo.piccinini@enea.it (M.P.); aurora.vincenti@enea.it (M.A.V.); alessandro.ampollini@enea.it (A.A.); paolo.nenzi@enea.it (P.N.); concetta.ronsivalle@enea.it (C.R.); 2 Fusion and Technologies for Safety and Security Department, ENEA C.R. Casaccia, Via Anguillarese 301, 00123 Rome, Italy

**Keywords:** lithium fluoride, color centers, thin films, Bragg curve, Bragg peak, radiophotoluminescence, proton beams, radiation detectors

## Abstract

Lithium fluoride (LiF) crystals and thin films are utilized as radiation detectors for energy diagnostics of proton beams. This is achieved by analyzing the Bragg curves in LiF obtained by imaging the radiophotoluminescence of color centers created by protons. In LiF crystals, the Bragg peak depth increases superlinearly with the particle energy. A previous study has shown that, when 35 MeV protons impinge at grazing incidence onto LiF films deposited on Si(100) substrates, the Bragg peak in the films is located at the depth where it would be found in Si rather than in LiF due to multiple Coulomb scattering. In this paper, Monte Carlo simulations of proton irradiations in the 1–8 MeV energy range are performed and compared to experimental Bragg curves in optically transparent LiF films on Si(100) substrates. Our study focuses on this energy range because, as energy increases, the Bragg peak gradually shifts from the depth in LiF to that in Si. The impact of grazing incidence angle, LiF packing density, and film thickness on shaping the Bragg curve in the film is examined. At energies higher than 8 MeV, all these quantities must be considered, although the effect of packing density plays a minor role.

## 1. Introduction

Lithium fluoride (LiF) is a widely used radiation-sensitive inorganic material that exhibits excellent thermoluminescence, optically stimulated luminescence, and radiophotoluminescence (RPL) properties, making it an attractive material for radiation dosimetry and detection applications [1,2,3,4,5,6,7,8]. One of its unique features is the presence of stable color centers (CCs), which are point defects in the crystal lattice [9,10,11]. Some of these electronic defects absorb light and emit luminescence, such as the F_2_ and F_3_^+^ ones—two electrons bound to two and three anion vacancies, respectively—which emit visible light in the red and green spectral regions, respectively, when optically excited in their overlapping broad absorption bands, also known as the M band [12], which peaks at ~450 nm. The formation of CCs is influenced by various factors, such as radiation type and exposure, temperature, and the presence of impurities [13,14]. Understanding the properties of CCs in LiF is crucial for developing high-performance radiation detectors and dosimeters, as well as for studying radiation-induced damage in materials [15,16,17,18].

Thin films of LiF can be deposited onto different substrates for various applications [19,20]. While several growth techniques are available for the production of thin films, including sputtering and electrochemical deposition, suitable for ternary oxides [21] and alloys [22], simple physical vapor deposition (PVD) methods are appropriate for depositing homogeneous thin films of LiF, an alkali halide material with a band gap of ~14 eV, the widest among solids [19,20]. Both thermal evaporation [16,18,19] and electron-beam assisted PVD [23,24], which involve heating solid powder until it vaporizes and condenses onto a substrate, have been widely utilized for the growth of LiF films, whose stoichiometry is preserved [18,25] due to the high binding energy of LiF.

Silicon (Si) is an interesting substrate material for LiF thin films due to its compatibility with many electronic and optical devices [19,20]. The combination of LiF films and Si substrates has been studied for its potential use in photonic devices and radiation detectors. Reflective Si substrates offer the advantage of signal amplification of the visible photoluminescence emitted by the optically active CCs in the film. This is due to the reflection from the substrate of those photons that would otherwise not be detected by an optical system, such as a fluorescence microscope [19,26].

In recent years, investigations into the behavior of CCs in LiF induced by ion and proton irradiation have been the subject of many studies [16,17]. This is largely due to the growing interest in using LiF detectors based on RPL for ion beam track recording [27,28] and proton energy diagnostics [6,29,30]. One approach to achieving the latter aim is by recording the Bragg curve in LiF crystals or thin films in the form of a volume distribution of CCs created by the interaction with protons [29,31]. Since the volume concentration of CCs is point-by-point proportional to the energy that protons lose for absorbed doses up to ~10^5^ Gy [32], the RPL emitted by the optically active F_2_ and F_3_^+^ centers can be exploited to obtain an image translation of the spatial distribution of energy deposited in the material [33]. As this spatial distribution depends on the energy spectrum of the protons, it is possible to estimate this spectrum by analyzing the RPL image, also thanks to the high sensitivity and high spatial resolution across a large field of view of these detectors [6]. This technique has gained considerable attention due to its potential applications in a wide range of fields, including radiation dosimetry [30].

In a previous paper, we analyzed Bragg curves generated at grazing incidence by 35 MeV protons in a LiF film on a Si(100) substrate, demonstrating that the Bragg peak in the film is located at a depth corresponding to the one that would be found in the underlying substrate rather than in the film material [34]. We ascribed this phenomenon to multiple Coulomb scattering (MCS), causing the simultaneous depletion of protons from the film and the migration of protons from the substrate into the film. In the present paper, we study the formation of the Bragg curve in LiF films on Si substrates within the 1–8 MeV range of proton energies for grazing incidence. This energy range is of particular interest because the position of the Bragg peak, stored as the maximum density of CCs in the film, gradually shifts with increasing energy from its expected depth in LiF bulk to the one it would have in Si bulk. Various aspects related to the Bragg curves in the film for energies within the explored range are analyzed and discussed, and two experimental cases involving energies close to the extremes of the studied energy range are presented.

## 2. Materials and Methods

Polycrystalline LiF films of several thicknesses were deposited by thermal evaporation on Si(100) substrates in controlled experimental conditions, starting from LiF powder (Merck Suprapur, 99.99% pure) heated in a tantalum crucible at about 850 °C. The Si(100) substrates, cleaned using detergents in ultrasonic baths, were placed on a rotating sample-holder in the process steel vacuum chamber of the deposition system at 22 cm from the crucible. The pressure inside the chamber was below 1 mPa prior to the evaporations, while the substrate temperature was kept at 300 °C during the deposition processes. The deposition rate, fixed at the nominal value of 1 nm/s, and the film thickness were monitored in situ by an INFICON quartz-crystal oscillator.

The spectrophotometers utilized for optical reflectance measurements were a model Lambda 1050 and a model Lambda 900 by Perkin Elmer for sample A-twin and B-twin, respectively, both equipped with a specular-reflectance accessory.

The TOP-IMPLART linear accelerator (linac) is composed of a commercial injector, PL-7 by ACCSYS-HYTACHI, operating at 425 MHz, followed by a booster section operating at 2997.92 MHz. The injector includes a duoplasmatron 30 keV proton source followed by two accelerating structures, a radio-frequency quadrupole (RFQ) and a drift tube linac (DTL), which accelerate the proton beam to 3 and 7 MeV, respectively. The booster is a sequence of eight accelerating modules that raises the proton energy from 7 to 71 MeV. Between the injector and the booster section, a medium energy beam transfer line (MEBT), which includes four electromagnetic quadrupoles and a 90° bending magnet, provides either the focusing of the beam towards the horizontal line or its focusing and bending towards a vertical extraction line.

The proton beam propagates in a vacuum within the accelerator. There are two extraction points in air: a high-energy one (maximum energy of 71 MeV) on the horizontal line and a low-energy one (energy range of 3–7 MeV) on the vertical line. The vertical line was designed specifically for in vivo radiobiology to allow irradiation of horizontally placed samples. Presently, it is being employed for research on fluorescent nuclear track-detector dosimeters based on LiF crystals and hybrid LiF-polymeric microgel thin films (BIOTRACK project [35]). It is 80 cm long and includes a 2 µm gold foil followed by a 2 mm diameter collimator. The gold foil is used to obtain a round and homogeneous irradiation spot on the target. The beam line is operated in a vacuum, and the protons are extracted from the air through a 50-µm thick Kapton window. Beam parameters at the vertical line exit are summarized in Table 1.

The difference between the energy values at the injector output and at the line exit is due to the energy degradation of the beam when crossing the gold foil and the Kapton window. The 1 MeV energy is obtained from the 3 MeV beam produced by the RFQ when the DTL is switched off, and the 6 MeV energy is obtained from the 7 MeV beam produced by the RFQ and DTL operating at nominal accelerating electric fields. Intermediate energies can be produced by reducing the amplitude of the accelerating field in the DTL. The 90° bending magnet current is adjusted to allow transport of the desired beam energy.

The RPL measurements reported in this paper were performed on samples irradiated in two different configurations: one irradiation (sample B) occurred at the end of the MEBT during the commissioning of the injector, before mounting the first accelerating module of the booster section; the second irradiation (sample A) occurred at the exit of the vertical line. For this latter, the DTL was switched off, and the amplitude of the electric field in the RFQ was set to achieve an output beam energy of around 1 MeV.

**Figure 1:**
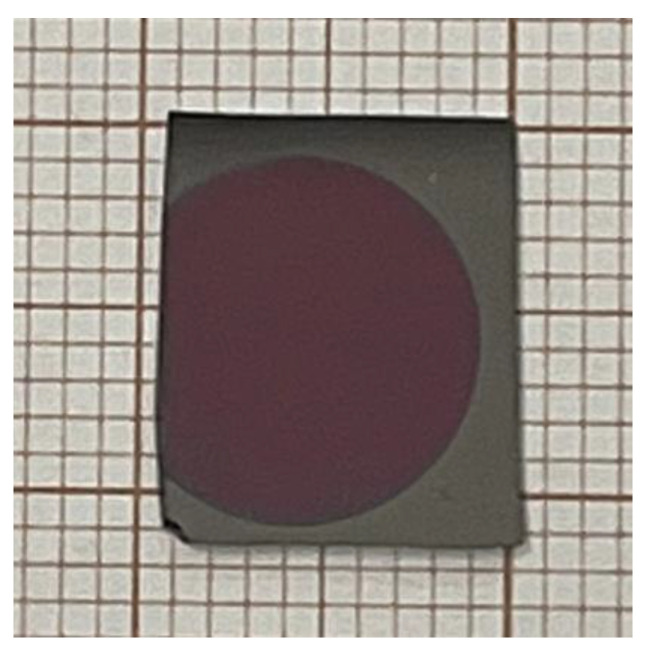
LiF film thermally evaporated on a Si(100) substrate and cleaved to obtain a clear-cut edge covered by the LiF layer. Background: graph paper.

**Figure 2:**
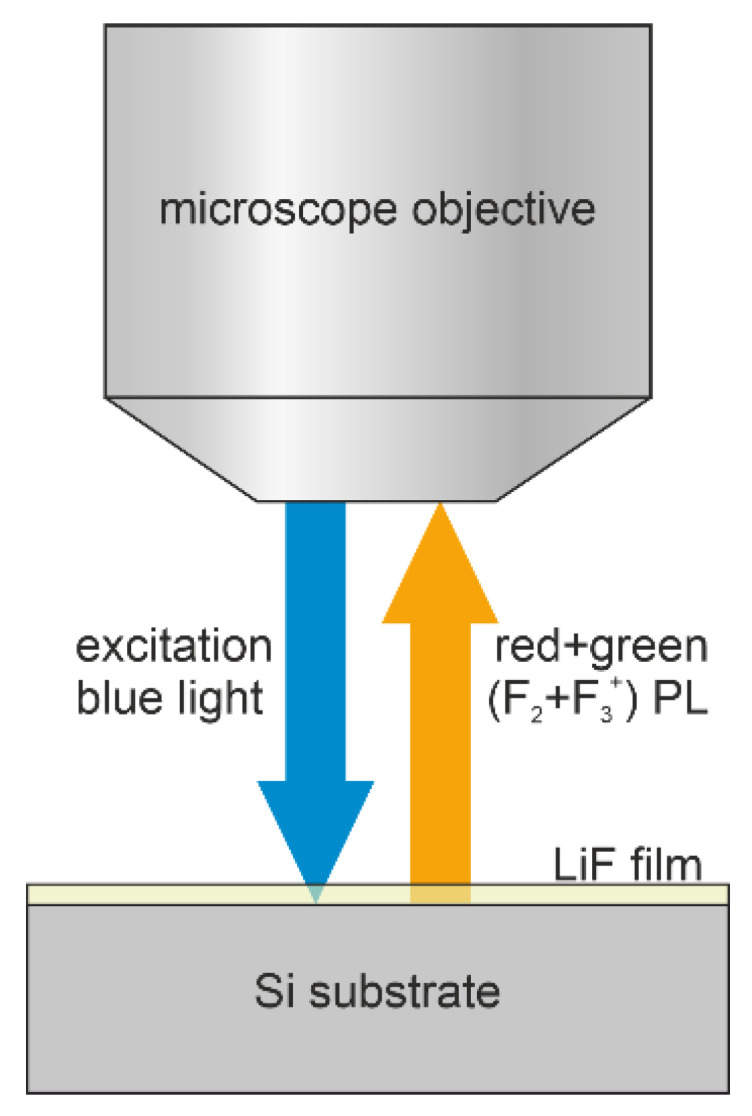
Simplified scheme (not to scale) of the fluorescence microscope setup used to image the RPL emitted by CCs formed in the LiF films under irradiation at grazing incidence with proton beams.

After the LiF film growth, the Si(100) substrates were cleaved into two parts to obtain a clear-cut edge covered by the LiF layer (Figure 1). They were mounted parallel to the beam propagation axis and with the cleaved edge perpendicularly facing the impinging proton beam to obtain a zero grazing incidence angle. For each sample, the fluorescence image acquired from the top surface side of the film allowed us to obtain the depth profile of the energy released by protons. In this irradiation geometry, the concentration of CCs vs. penetration depth is point by point proportional to the energy loss curve—that is, the Bragg curve—provided CC saturation is not reached [29,33,36,37,38]. The visible RPL Bragg curve images latently stored in the irradiated films, due to the F_2_ and F_3_^+^ CCs, were acquired by a Nikon 80-i fluorescence microscope equipped with an Andor NEO s-CMOS camera and a Hg-lamp, whose emission was properly filtered in the blue region to simultaneously excite the RPL of these CCs in their absorption band (Figure 2). The broad red and green emissions from the F_2_ and F_3_^+^ centers were spectrally integrated at wavelengths above 520 nm to obtain a good enough signal-to-noise ratio despite the film’s low thickness. Experimental Bragg curve profiles were obtained by summing the RPL pixel intensity transversally to the proton beam propagation axis using ImageJ software (version 1.47) [39] within a region of interest (ROI) selected in the images.

The Monte Carlo simulations were performed using FLUKA version 4-3.1 software [40,41,42] and its graphic interface, Flair version 3.2-2 [43].

## 3. Results

Before reporting the experimental results concerning characterization and RPL imaging of the proton-irradiated samples under investigation, we show here a few Monte Carlo simulations, performed in FLUKA, of energy deposition in LiF thin films on Si substrates irradiated at grazing incidence by protons of energies from 1 to 8 MeV. The purpose of these simulations is twofold: first, it is a convenient way to anticipate what we should expect from the real cases analyzed afterwards; second, the explored energy range is of great interest because it is where, for increasing energies, the Bragg peak in the LiF film shifts from its expected position to a deeper position corresponding to the maximum of energy deposition in the underlying substrate.

### 3.1. Monte Carlo Simulations

In a previous study, it was demonstrated through both experimental measurements and Monte Carlo simulations that the depth of the Bragg peak in a LiF thin film on a Si substrate, induced by a 35 MeV proton beam incident at grazing angle, was located at a depth corresponding to that of Si rather than LiF [34]. This was attributed to MCS, which caused both the leakage of protons that initially entered the film and the migration of protons from the substrate into the film. As a result, the protons stopping in the film and contributing to the formation of the Bragg peak in it had traveled most of their paths in Si. This implies that a LiF film can serve as an information transducer for visualizing the Bragg peak depth in the underlying substrate through the stable formation of CCs.

**Figure 3:**
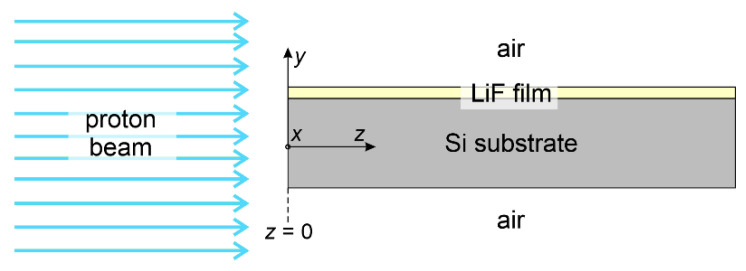
Simplified scheme (not to scale) of the setup considered in FLUKA for the energy deposition simulations. The *z* axis lies along the propagation direction of the proton beam. The *x* axis is perpendicular to the page. The *y* axis lies along the sample thickness and is perpendicular to both the *x* and the *z* axes. The sample’s cleaved edge facing the incoming beam is at *z* = 0.

**Figure 4:**
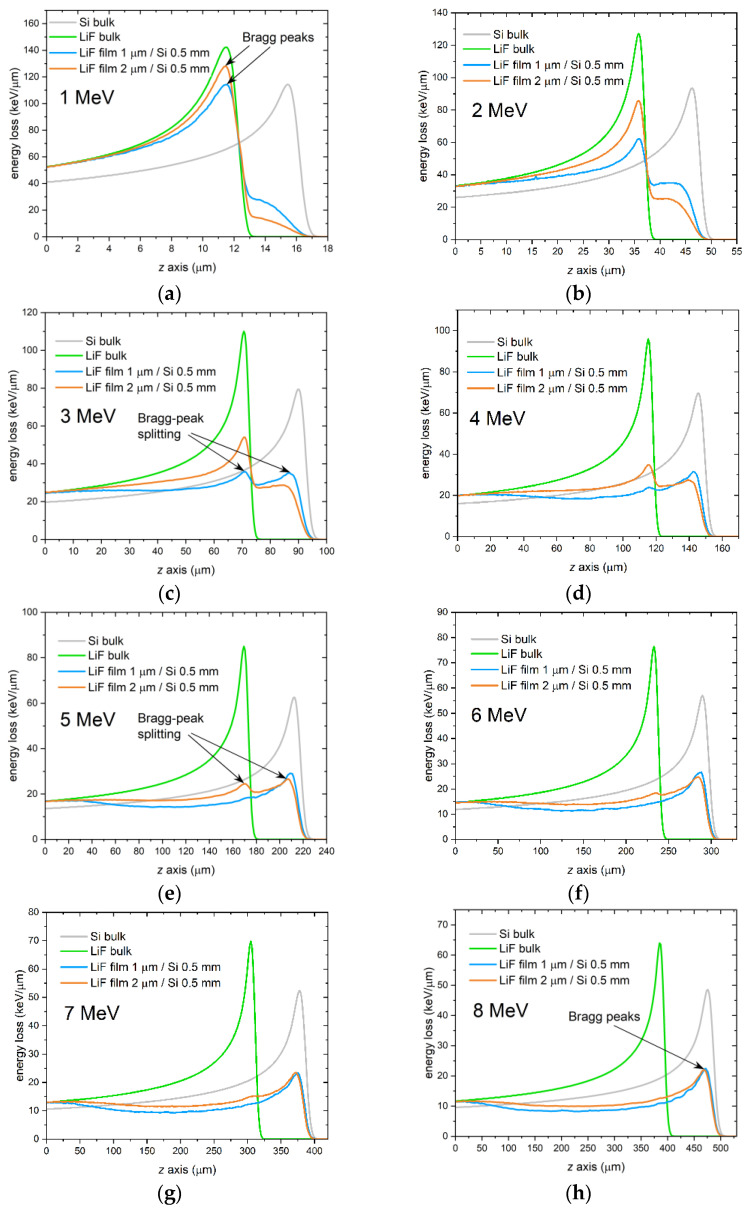
Simulated energy-loss curves vs. penetration depth (*z* axis) for monochromatic proton beams impinging onto a Si bulk (gray lines), a LiF bulk (green lines), and at zero grazing incidence angle onto a 1-µm thick (blue lines) and a 2-µm thick (orange lines) LiF film on a 0.5-mm thick Si substrate. Proton energies: (**a**) 1 MeV; (**b**) 2 MeV; (**c**) 3 MeV; (**d**) 4 MeV; (**e**) 5 MeV; (**f**) 6 MeV; (**g**) 7 MeV; (**h**) 8 MeV.

The purpose of the Monte Carlo simulations presented in this paper is to investigate the range of proton energies within which the depth of the Bragg peak in LiF films on Si substrates gradually shifts from the expected depth in LiF to what it would be in Si. A simplified scheme of the setup used in the simulations is shown in Figure 3. The simulation results indicate that the Bragg peak shift indeed occurs within the energy range of 1 to 8 MeV for LiF films with a thickness of about 1 to 2 µm on a Si substrate, as shown in Figure 4. The figure displays FLUKA simulations of energy-loss curves in 1 µm and 2 µm LiF films on 0.5-mm thick Si substrates, produced by monochromatic proton beams incident at zero grazing angle with energies ranging from 1 to 8 MeV in increments of 1 MeV. Simulated energy-loss curves for LiF bulk (density of 2.635 g/cm^3^ [44]) and Si bulk (density of 2.33 g/cm^3^ [45]) are also included to show their Bragg peak positions. Note that the depth of the Bragg peak in Si bulk is always deeper than in LiF bulk due to the lower density of Si. In the simulations, the beam fluences were 10^8^ protons/cm^2^ for the films and 10^6^ protons/cm^2^ for the bulks. It can be clearly seen in Figure 4 that at the lowest energy of 1 MeV, the Bragg peak depth in the LiF film coincides with that in the LiF bulk, while the beginning of a deeper contribution is visible. At an energy of 3 MeV, this latter starts to take the shape of a peak located at the depth it would be found in Si bulk, and up to 5 MeV, it coexists with the original peak corresponding to the depth in LiF bulk, as if the peak had split into two. Above 5 MeV, the peak corresponding to the Bragg peak in Si bulk becomes predominant, and the peak at the LiF-bulk depth practically disappears from 6–7 MeV onwards.

As previously mentioned, the shift in the Bragg peak depth is attributed to two concomitant effects of MCS: the leakage of protons from the film and the migration of protons from the substrate into the film. When the energy is high enough that all the protons that have entered the film have left it after being scattered multiple times in the LiF lattice, a large reservoir of protons is available to enter the film from the substrate due to the much greater thickness of the substrate compared to that of the film. Although some protons also enter the film from the air side, the low density of air results in much fewer scattering events and a much lower frequency of proton entry. Thus, a gradual shift of the Bragg peak from the LiF to the Si position is detected as proton energy increases.

**Figure 5:**
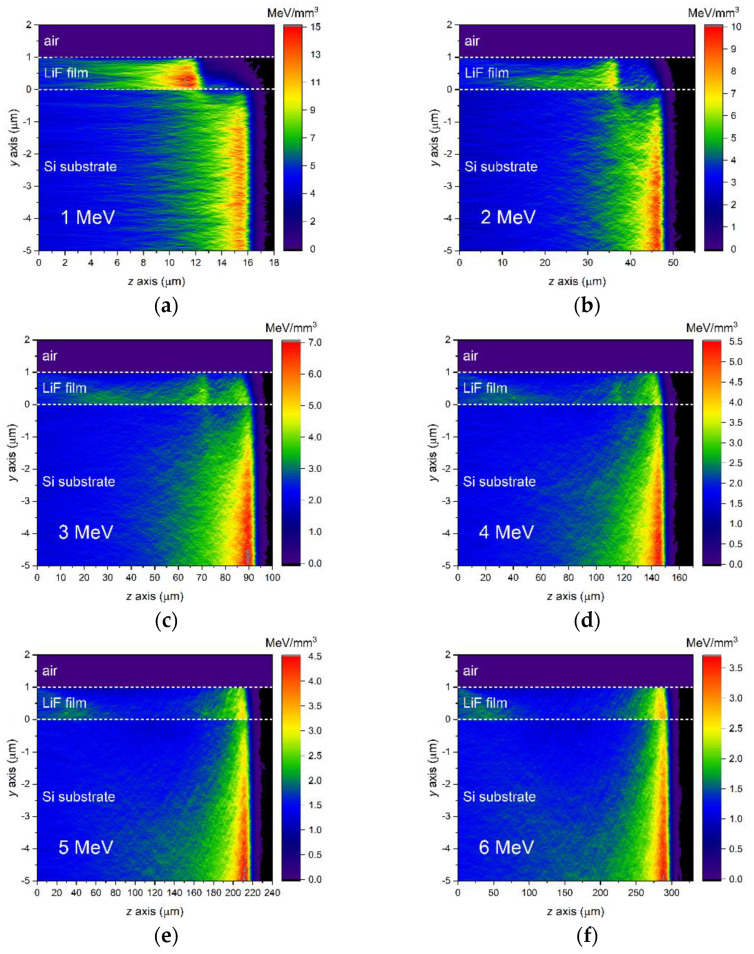

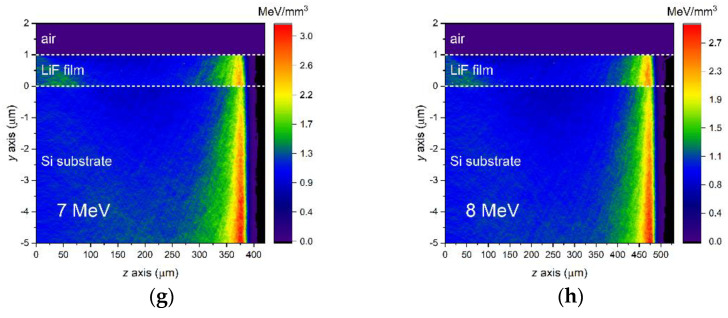
Simulated *x*-projected 2D maps of energy density per proton deposited in the LiF film and in the Si substrate by monochromatic proton beams impinging at a zero grazing incidence angle. Proton energies: (**a**) 1 MeV; (**b**) 2 MeV; (**c**) 3 MeV; (**d**) 4 MeV; (**e**) 5 MeV; (**f**) 6 MeV; (**g**) 7 MeV; (**h**) 8 MeV.

In addition to the curves shown in Figure 4, the shift can also be clearly visualized by observing the 2D projection maps of energy loss displayed in Figure 5. These maps, which are only shown for the 1-µm thick film for the sake of brevity, are projections along *x* of the energy density per proton that is deposited in the sample. The projections are averages over the *x*-width of the virtual detector in FLUKA. Since this x-width is set to be much larger than the impinging beam width to enclose all the scattered protons, the indicated units (MeV/mm^3^) should be considered relative rather than absolute. It is clearly visible how the depths of the maximum energy densities in the film and in the substrate gradually become closer and closer as energy increases, and that during this transition, the energy deposited in the film features two high density zones.

### 3.2. Bragg Curves in LiF Films Thermally Evaporated on Si Substrates

The previous section presented Monte Carlo simulations that predict a gradual shift of the Bragg peak in a LiF film for proton beams with energies changing from 1 to 8 MeV. The shift occurs such that at the lowest energies, the Bragg peak is located where it would be expected in LiF bulk, while at the highest energies, it moves to depths that coincide with those that would be found in Si rather than LiF. In the following, we present two experimental cases that show the predicted behavior in real LiF films. After irradiation with a proton beam, the intensity of the RPL emitted by the CCs created in the LiF films, due to the interaction of the crystalline material with the protons, is directly proportional to the energy deposited by the protons. As a result, detecting the RPL image latently stored in the film enables the reconstruction of a replica of the Bragg curve in the film material.

#### 3.2.1. Thin Film Deposition

The two LiF films we considered are here referred to as A and B. Both films were deposited on Si(100) substrates of thickness 0.5 mm. The nominal thicknesses of the films were 1.5 µm and 1 µm for A and B, respectively.

#### 3.2.2. Optical Characterization

**Figure 6:**
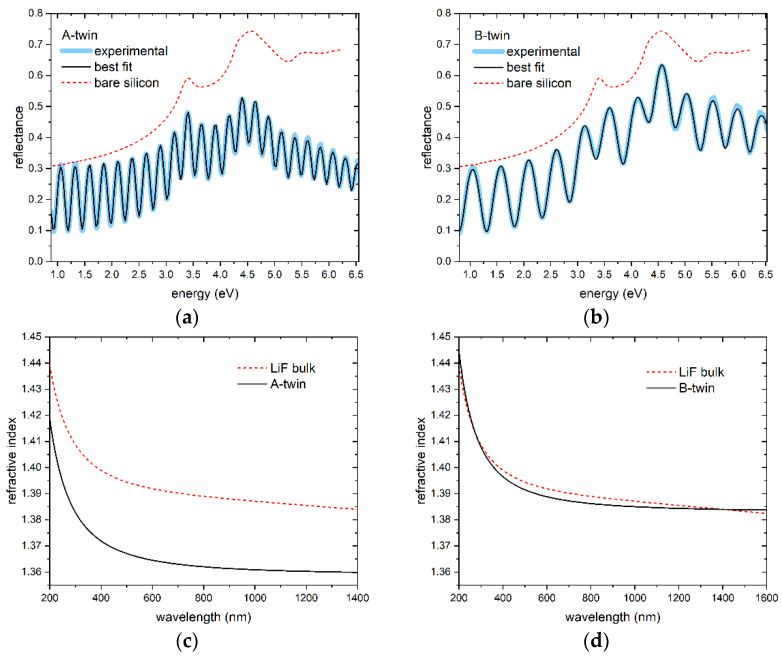
Experimental (thick blue solid lines) and best-fitting (black solid lines) reflectance spectra: (**a**) sample A-twin; (**b**) sample B-twin. Reflectance spectra of bare Si (red dashed lines) are also shown for comparison. Corresponding refractive-index dispersions of the films (black solid lines) compared with those of LiF bulk (red dashed lines) [47]: (**c**) sample A-twin; (**d**) sample B-twin.

The measured specular reflectance spectra of two films, named A-twin and B-twin, obtained under identical conditions as films A and B in the same deposition runs, respectively, are shown in Figure 6 along with their corresponding refractive-index dispersions. To obtain these dispersions, an accurate best-fit model [46,47] was utilized, which also allowed the estimation of the thickness of the films. Specifically, the thickness values of A-twin and B-twin were determined to be ~1.72 µm and ~585 nm, respectively. By comparing the obtained refractive index dispersions with that of LiF bulk [47], which is also shown in Figure 6, packing density values of ~93.3% and ~99.5% were estimated for A-twin and B-twin, respectively. For the subsequent analysis, it is assumed that the same thickness and packing density values of A-twin can be used for A, and similarly, the same values of B-twin can be used for B.

#### 3.2.3. Bragg Curve Measurement and Analysis

Sample A was irradiated at the vertical beamline of the TOP-IMPLART accelerator with a proton beam of nominal energy 3 MeV at a dose of 4 × 10^3^ Gy delivered at the LiF-air interface in 150 s. Sample B was irradiated at the horizontal beamline with a proton beam of nominal energy 7 MeV at a dose of 3.4 × 10^4^ Gy delivered at the LiF-air interface in 20 s.

As already discussed in the Introduction, the irradiation of LiF with ionizing radiation induces the formation of CCs in the material, some of which—the F_2_ and F_3_^+^ ones—emit red and green RPL, respectively, when optically excited with blue light in the M absorption band. In the case of protons, the RPL intensity emitted by F_2_ and F_3_^+^ CCs in LiF films has been found to be directly proportional to the absorbed dose within a broad range of doses [32,37]. As explained in the Section 2, a rectangular ROI was used to extract an RPL intensity profile corresponding to the Bragg curve from the images of the irradiated samples acquired by the fluorescence microscope.

**Figure 7:**
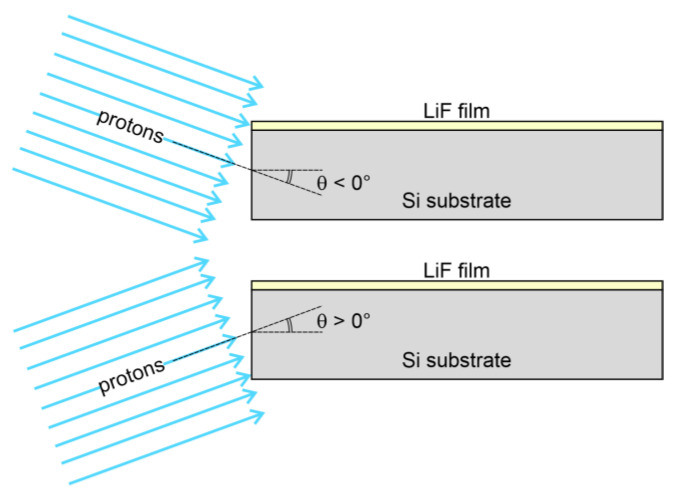
Grazing incidence angle sign convention. A non-zero grazing incidence angle θ is considered negative when the proton beam originates from the half space above the LiF film and positive when the proton beam originates from the half space below the film (substrate side).

The experimental RPL intensity profiles of the two films were compared to FLUKA simulations of energy deposition in the films. The simulations were fine-tuned by adjusting a few parameters, including the irradiation geometry and the shape of the energy spectrum of the beam, which was assumed to consist of a sum of Gaussian components for simplicity. The grazing incidence angle θ was found to be a critical parameter for accurate reproduction of the full experimental profiles. As discussed in [34], this angle—whose sign convention is explained in Figure 7—not only affects the overall shape of the RPL intensity profile but also causes the formation of CCs in the film at distances *z* larger than the detected Bragg peak depth when it is negative. There, the concentration of CCs—and consequently the intensity of the emitted RPL—significantly depends on even small variations of the value of θ. Another important quantity to consider in the simulations is the packing density of the LiF films. Indeed, the range of energetic ions is inversely proportional to the material density [48], meaning that a lower packing density will result in a longer range and a Bragg peak in the film that is further from the sample edge. However, we expect the influence of the LiF packing density on the Bragg peak position in the film to be negligible at those proton energies—e.g., larger than about 5 MeV—for which the Bragg peak formation is mostly due to protons that migrated from the substrate into the film (see Figure 4).

**Figure 8:**
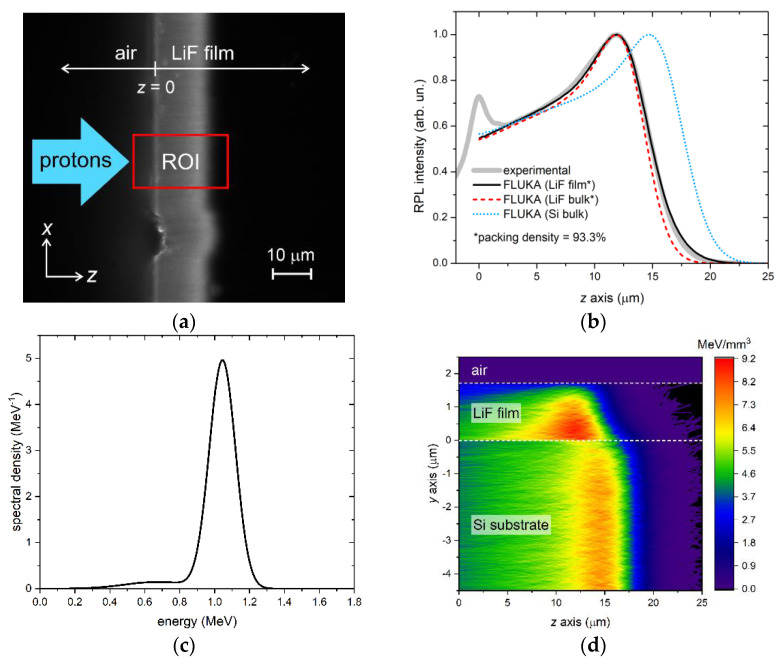
Measured RPL fluorescence image and FLUKA simulation of LiF film A. (**a**) RPL image detected at the fluorescence microscope—the red rectangle labeled ROI is the region of interest from which the experimental RPL intensity profile vs. depth in (**b**) is extracted; (**b**) RPL intensity profile vs. depth (gray thick solid line) extracted from the RPL image ROI in (**a**) and FLUKA simulations of RPL intensity (set proportional to energy loss) in the LiF film on Si (black solid line), a LiF bulk with the same packing density as that of the film (red dashed line), a Si bulk (blue dotted line); (**c**) energy spectrum corresponding to the FLUKA simulations in (**b**); (**d**) *x*-projected 2D map of the energy density per proton deposited in the top part of the LiF film on a Si sample.

The first case considered regards film A. This sample was irradiated with a proton beam with a nominal energy of 3 MeV. The RPL image, detected by the fluorescence microscope with a 100× objective after the irradiation, is reported in Figure 8a, where the rectangular ROI is shown in red. For each depth value *z*, the RPL intensity profile was calculated by summing along the *x* axis the intensities of the pixels enclosed in the ROI. The resulting profile (after normalization) is plotted in Figure 8b, together with the normalized FLUKA simulation obtained with the energy spectrum of Figure 8c, with a fluence of 3 × 10^8^ protons/cm^2^ and a grazing incidence angle θ = +0.50°. The simulated curve closely matches the experimental profile, except for a small peak at *z* = 0 in the experimental data, which is likely caused by light scattering at the sample edge and is not present in the simulation. The energy spectrum corresponding to the simulation consists of a main Gaussian peak centered at ~1.05 MeV plus a lower-energy tail realized with a broad, less intense (7% of the total energy) Gaussian component centered at ~0.68 MeV. In the simulation, the 93.3% packing density of the film material, as estimated from the optical characterization of A-twin, was duly considered. It should be noted that the packing density of a material influences the penetration of accelerated protons into it. Indeed, because the Bragg-Kleeman rule states that the range of energetic ions is inversely proportional to the material density [48], lighter materials with voids or air interstitials result in a longer propagation of protons. For comparison, Figure 8b also shows simulations of normalized energy deposition in LiF bulk (with packing density set equal to that of the LiF film) and in Si bulk. As expected from the simulations reported in Section 3.1, at these low energies, the Bragg peak in the LiF film is found at the same depth as it would be found in the LiF bulk, while the peak in the Si bulk is located at a deeper depth due to the lower density of Si with respect to LiF. Finally, Figure 8d shows a 2D projection of the energy density per proton deposited in the top part of the sample, which includes the LiF film and 4.5 µm of Si. As for Figure 5, this projection is an average over the *x*-width of the virtual detector in FLUKA. Since this x-width is set to be much larger than the impinging beam width to enclose all the scattered protons, the indicated units (MeV/mm^3^) should be considered relative rather than absolute.

**Figure 9:**
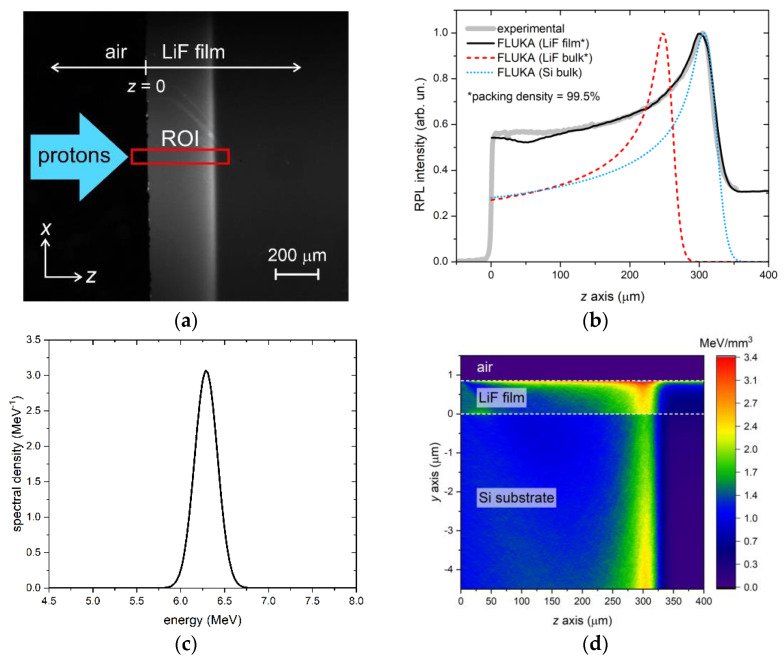
Measured RPL fluorescence image and FLUKA simulation of LiF film B. (**a**) RPL image detected at the fluorescence microscope—the red rectangle labeled ROI is the region of interest from which the experimental RPL intensity profile vs. depth in (**b**) is extracted; (**b**) RPL intensity profile vs. depth (gray thick solid line) extracted from the RPL image ROI in (**a**) and FLUKA simulations of RPL intensity (set proportional to energy loss) in the LiF film on Si (black solid line), a LiF bulk with the same packing density as that of the film (red dashed line), a Si bulk (blue dotted line); (**c**) energy spectrum corresponding to the FLUKA simulations in (**b**); (**d**) *x*-projected 2D map of the energy density per proton deposited in the top part of the LiF film on a Si sample.

The results for film B are presented in Figure 9. The four panels of this figure have the same meaning as those in Figure 8. The RPL intensity profile, obtained from the ROI of the RPL image detected at the fluorescence microscope with a 4× objective, is satisfactorily reproduced by simulations for this sample as well. In this case, the grazing incidence angle of the FLUKA simulation, performed with a fluence of 5 × 10^8^ protons/cm^2^, needed to be negative, θ = −0.41°, to accurately reproduce the full experimental profile, in line with the non-zero level of the RPL intensity detected beyond the Bragg peak depth. The sample was irradiated at the exit of the MEBT, ending with a 50 µm Kapton window, with the injector in the commissioning phase and not yet operating at maximum power. The energy spectrum derived from the simulation features a single Gaussian peak centered at ~6.29 MeV, as shown in Figure 9c. Taking into account the energy loss in the Kapton window, the measured value corresponds to an energy at the injector output in a vacuum of ~6.7 MeV, slightly lower than the nominal energy of 7 MeV. Consistent with the simulations in Section 3.1, the peak of the film RPL profile in Figure 9b coincides with that of the maximum energy deposition in Si bulk rather than in LiF bulk, due to the leakage of protons from the film and the simultaneous migration of protons from the substrate into the film caused by MCS. The equal depth of peaks in the film and in the substrate is also clearly visible in Figure 9d.

## 4. Discussion

The analysis of the experimental RPL profiles—due to the F_2_ and F_3_^+^ CCs created in the LiF films on Si(100) by proton irradiation—was carried out by comparing them with Monte Carlo simulations. This analysis revealed that the grazing incidence angle is a critical factor in determining certain features of the Bragg curve. Other characteristics that have the potential to affect the LiF film’s response are its thickness and packing density.

Understanding how main film characteristics, related to growth conditions, influence a film’s response to proton irradiation is crucial for its reliable use as a radiation detector for beam energy diagnostics by Bragg curve recording. The film growth conditions described in Section 2 were chosen based on previous investigations [19,26]. It is important to note that the present study does not aim to determine the optimal deposition parameters of LiF thin films regarding their structural and morphological properties or to investigate their effect on CC formation at a microscopic scale. Furthermore, while the presence of impurities cannot be completely ruled out, their effects on the formation, stabilization, and spectral features of the broad emission bands of the CCs can be considered negligible, given the excellent agreement between the experimental results and the simulations.

Considering the significant influence of both the grazing incidence angle and some film characteristics, we performed additional simulations in FLUKA to investigate the energy deposition of accelerated protons in LiF films on Si substrates at the two energy extremes previously examined in Section 3.1. This was performed while varying the grazing incidence angle, film packing density, and thickness.

### 4.1. Role of the Grazing Incidence Angle

In the proton irradiation stage, the samples were carefully aligned as parallel as possible with respect to the beam direction to achieve a zero grazing incidence angle, i.e., θ = 0°. However, the simulations suggest that the actual grazing angles were not zero, with θ = +0.50° and θ = −0.41° for films A and B, respectively. To better understand the degree of misalignment that these angles imply, one can consider that a tilt of ±0.5° for a 1-cm-long sample corresponds to a height difference of approximately 87 µm between the front and back edges of it, which is smaller than the thickness of standard copy paper (~100 µm for 80 gsm paper). Achieving perfect alignment between sample and beam with a precision smaller than this value can be a challenging task. Hence, in principle, tilts within ±0.5° could be considered good enough. However, such small angular values have a greater effect on the shape of the Bragg curve in the film than one might expect. In particular, for the examined cases, the most visible effect of non-zero grazing incidence angles is on the tail beyond the Bragg peak. Indeed, observing Figure 8b, one can see that for θ > 0°, there is no RPL contribution deeper than the Bragg peak, as otherwise seen for θ = 0° in Figure 4a. On the other hand, Figure 9b demonstrates that for θ < 0°, the curve tail beyond the Bragg peak forms an evident plateau due to protons arriving into the film from the air side above it, which is not present in the simulation at θ = 0° in Figure 4f.

**Figure 10:**
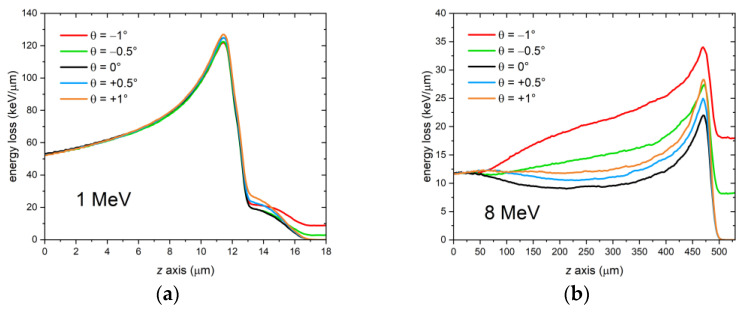
FLUKA simulations of the energy deposited per proton in a 1.5-μm thick LiF film on a 0.5-mm thick Si substrate by a monochromatic proton beam impinging with different grazing incidence angles θ. Proton beam energy: (**a**) 1 MeV; (**b**) 8 MeV.

More generally, changes in the grazing incidence angle can cause significant alterations in the overall shape of the Bragg curve within the film, particularly at higher energies in the investigated 1–8 MeV range. To demonstrate the extent of these changes, we simulated a 1.5-μm thick LiF film on a 0.5-mm thick Si substrate exposed to a monochromatic proton beam (simulation energies: 1 MeV and 8 MeV) and compared cases with different values of the grazing incidence angle θ, including zero, positive, and negative angles. The external medium was air, as in the previous simulations, and the fluence was 10^8^ protons/cm^2^. The film’s packing density was 100%. The resulting curves are shown in Figure 10. While changes in the Bragg curve at 1 MeV are almost negligible, it is evident that at 8 MeV the effect on the Bragg curve shape is quite dramatic, especially for θ < 0°. As higher energy leads to more scattering events in both the film and substrate, one can conclude that the impact of this angle on the Bragg curve shape is amplified by MCS. Notably, the position of the Bragg peak remains practically the same for the different grazing incidence angles at both energies.

### 4.2. Role of the Film Packing Density

**Figure 11:**
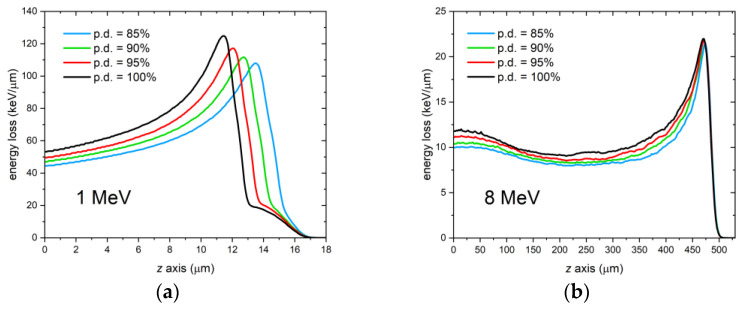
FLUKA simulations of the energy deposited per proton in 1.5-μm thick LiF films with different packing densities (p.d.) on a 0.5-mm thick Si substrate by a monochromatic proton beam impinging at perfect grazing incidence θ = 0°. Proton beam energy: (**a**) 1 MeV; (**b**) 8 MeV.

As already stated, the packing density of a material influences the penetration of accelerated protons into it. Indeed, because the Bragg-Kleeman rule states that the range of energetic ions is inversely proportional to the material density [48], lighter materials with voids or air interstitials result in a longer propagation of protons. While this is certainly true for bulk, the case of a thin film on a substrate could be slightly different. To understand the role of packing density in the case of a thin film, we simulated proton irradiations at 1 MeV and 8 MeV of a 1.5-μm thick LiF film on a 0.5-mm thick Si substrate using different values of the film packing density from 85% to 100%. As in the previous simulations, the external medium was air, and the fluence was 10^8^ protons/cm^2^. The grazing incidence angle was θ = 0°. Figure 11 shows the curves resulting from the simulations. It can be observed that there is a clear shift of the Bragg peak at 1 MeV, whereas no shift is present at 8 MeV. This is because at 1 MeV, the Bragg peak is due to protons that have traveled mostly in the film and whose range is affected by the density of LiF. In contrast, at 8 MeV, the Bragg peak is primarily caused by protons that have traveled most of their path in the underlying Si substrate and are only marginally influenced by changes in the density of the film material.

### 4.3. Role of the Film Thickness

**Figure 12:**
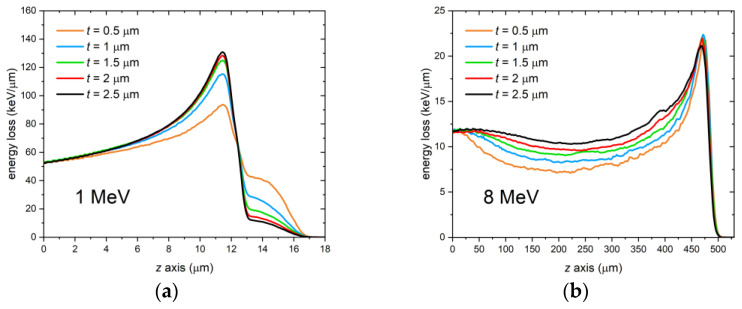
FLUKA simulations of the energy deposited per proton in LiF films of different thicknesses *t* on a 0.5-mm thick Si substrate by a monochromatic proton beam impinging at perfect grazing incidence θ = 0°. Proton beam energy: (**a**) 1 MeV; (**b**) 8 MeV.

Another parameter worth evaluating for its effect on the Bragg curve in the LiF film is the film thickness. Monte Carlo simulations were run for this case, at grazing incidence angle θ = 0° and LiF packing density of 100%, for a few different thickness values between 0.5 µm and 2.5 µm of the film on a 0.5-mm thick Si substrate. Again, two simulation energies were selected corresponding to the extremes of the investigated energy range (1 MeV and 8 MeV); the external medium was air, and the fluence was 10^8^ protons/cm^2^ (2 × 10^8^ protons/cm^2^ just for the 0.5-µm thick film at 8 MeV to limit statistical noise and obtain a smoother curve). The resulting Bragg curves are shown in Figure 12. For both energies, a thicker film results in less depletion of protons from it and a relatively smaller contribution to the Bragg curve of protons arriving from the substrate, as one would expect.

## 5. Conclusions

Imaging the visible RPL emitted by stable CCs created by the interaction of protons with the LiF crystal lattice and analyzing it using Bragg curve models and Monte Carlo simulations is an established technique for proton beam energy diagnostics using solid-state LiF-based radiation detectors. In optically transparent thin-film LiF detectors thermally evaporated on Si(100) and for a 35 MeV proton beam, it was observed that the Bragg curve formation in the film is mainly due to protons migrating into the film from the underlying substrate as a result of MCS [34]. For this reason, the depth of the Bragg peak in the film corresponds to what one would find in the substrate material rather than in LiF.

In this paper, we have considered LiF thin films on Si substrates and investigated the energy range 1–8 MeV within which a gradual shift of the Bragg peak occurs from the depth it has in LiF to the one that would be found in Si. Both Monte Carlo simulations and measured RPL profiles, acquired with a fluorescence microscope, were used to conduct the study. The results show that the grazing incidence angle significantly shapes the Bragg curve in the LiF film at the highest investigated energies. Meanwhile, the packing density of the film determines the depth of the Bragg peak at the lowest energies, with no significant effects at the highest energies. On the other hand, the film thickness influences the shaping of the Bragg curve at both extremes of the investigated energy range. These findings suggest that while the packing density of LiF thin-film detectors may not significantly affect the shaping of the Bragg curve of proton beams at higher energies than those investigated in this study, such as those in the range of dozens of MeV planned for the TOP-IMPLART accelerator, it is crucial to consider the impact of the grazing incidence angle and film thickness. It is worth pointing out that, for such energies, the simulations also suggest that the depth of the Bragg peak is likely to be totally unaffected by changes in grazing incidence angle, LiF packing density, and film thickness.

## Figures and Tables

**Table 1:** Beam parameters at the linac vertical line exit.

Parameter	Value
Pulse length	15 to 60 µs
Pulse repetition frequency	25 Hz
Flux per pulse	10^6^ to 2 × 10^7^ p/cm^2^/pulse
Energy from the injector	3–7 MeV
Energy on target	1–6 MeV
Energy spread on target ^1^	80–90 keV
Maximum beam diameter on target	16 mm
Transverse homogeneity on target	±5%

^1^ Root-mean-square value for all the energies.

## Data Availability

The data presented in this study are available on request from the corresponding author.
